# Selective Microbial Genomic DNA Isolation Using Restriction Endonucleases

**DOI:** 10.1371/journal.pone.0109061

**Published:** 2014-10-03

**Authors:** Helen E. Barnes, Guohong Liu, Christopher Q. Weston, Paula King, Long K. Pham, Shannon Waltz, Kimberly T. Helzer, Laura Day, Dan Sphar, Robert T. Yamamoto, R. Allyn Forsyth

**Affiliations:** FLIR Systems, Inc., La Jolla, California, United States of America; The Scripps Research Institute and Sorrento Therapeutics, Inc., United States of America

## Abstract

To improve the metagenomic analysis of complex microbiomes, we have repurposed restriction endonucleases as methyl specific DNA binding proteins. As an example, we use DpnI immobilized on magnetic beads. The ten minute extraction technique allows specific binding of genomes containing the DpnI G^m6^ATC motif common in the genomic DNA of many bacteria including γ-proteobacteria. Using synthetic genome mixtures, we demonstrate 80% recovery of *Escherichia coli* genomic DNA even when only femtogram quantities are spiked into 10 µg of human DNA background. Binding is very specific with less than 0.5% of human DNA bound. Next Generation Sequencing of input and enriched synthetic mixtures results in over 100-fold enrichment of target genomes relative to human and plant DNA. We also show comparable enrichment when sequencing complex microbiomes such as those from creek water and human saliva. The technique can be broadened to other restriction enzymes allowing for the selective enrichment of trace and unculturable organisms from complex microbiomes and the stratification of organisms according to restriction enzyme enrichment.

## Introduction

Next Generation Sequencing (NGS) has reinvigorated the understanding of the role that bacteria play as symbionts and pathogens of plants [1], insects [2], vertebrates [3] and in the environment [4], [5]. NGS has broadened the study of the prokaryotic world beyond the small fraction of bacteria (less than 1%) thought to be culturable [6], [7], [8]. Using NGS for metagenomic studies, in which an entire sample of mixed organismal DNA is sequenced, has the advantage of querying the entire population of isolated DNA and overcomes many biases of other metagenomic methods such as microarray analysis or multiplex PCR. However, there are some drawbacks to using NGS metagenomic strategies. First, sensitivity to microbes may be decreased in the presence of large amounts of non-informative DNA (e.g. eukaryotic DNA). Second, typical metagenomic samples can contain hundreds of bacterial species making it difficult to parse and assemble genomes [9].

Recently developed methods to selectively enrich prokaryotic DNA exploit the 5-methylcytosine (5mC) in CpG sites of eukaryotes (mCpG), a modification largely absent in the bacterial world. One method uses a methyl-binding protein/Fc fusion protein to bind eukaryotic mCpG containing DNA and remove it from the mixture [10]. In an alternate approach, a truncated version of the human cytidylate-phosphate-deoxyguanylate protein has been used to bind non-methylated CpG sequences in bacterial DNA [11]. Bacteria have other stable epigenetic modifications in addition to 5mC including 6-methyladenine (6 mA) and 4-methylcytosine (4mC). The 6 mA modification was shown to occur at 94.1% of the 41,791 GATC sites in the *Escherichia coli* genome [12] and is widespread in prokaryotes but is otherwise reported only in ciliates and lower eukaryotes [13]. The DNA adenine methyltransferase (DamMT) directs adenine methylation within the context of GATC sequences and is found in at least one clade of bacteria consisting of the orders Enterobacteriales, Vibrionales, Aeromonadales, Pasteurellales and Alteromonadales [14]. In *E. coli*, GATC methylation influences chromosome replication, gene expression and mismatch repair. In *Vibrio cholerae* it is required for viability and in *Salmonella enterica* and *Haemophilus influenzae* it may act as a virulence factor [14]. 6 mA is also generated by some methyltransferases (MTases) as part of restriction modification systems [15]. Restriction endonucleases rely on methylation patterns to combat invasive genomes, particularly phage, while avoiding digestion of host DNA. Evolution has thus selected for enzymes with exquisite methylation sensitivity.

Here we present a restriction endonuclease-mediated DNA enrichment approach. DpnI is a methyl-directed restriction endonuclease that restricts DNA only when it is methylated on adenine residues within the GATC sequence [16], [17]. We therefore anticipated that DpnI could distinguish bacterial genomes containing the G^m6^ATC DNA modification from other bacterial and eukaryotic DNA. By manipulating the reaction conditions, we can use it to bind DNA without cutting. Since DpnI binds to DNA only when it is adenine methylated within GATC sites we predicted little or no binding to eukaryotic DNA and highly specific binding to DNA from DamMT+ bacteria. We demonstrate that DpnI can selectively enrich microbial DNA from synthetic and real-world samples. We extend our approach to a second restriction enzyme, DpnII that specifically enriches non-methylated GATC DNA (*e.g*. human genome). DNA enriched by this method can be used for PCR, qPCR and NGS analysis. The technique can enable the targeted enrichment of genomes from various microbiomes or the specific identification of pathogens from complex samples. We envision the use of restriction endonuclease binders to stratify complex metagenomic samples into groupings based on methylome signatures. This could link DNA fragments in otherwise poorly assembled contigs, aiding the reconstruction of genomes from unculturable organisms.

## Materials and Methods

Genomic DNA was obtained from the ATCC with the exception of the following: *E. coli* K12 (Affymetrix, Santa Clara, CA); *Yersinia pestis*, *Franscisella tularensis*, *Burkholderia mallei*, *Burkholderia cepacia*, *Brucella abortus*, *Bacillus anthracis* (BEI Resources, Manassas, VA); and Human, Arabidopsis and Rice (Zyagen, San Diego, CA). Commercially available DpnI and pUC19 were purchased from NEB (Ipswich, MA).

### DpnI purification and biotinylation

DpnI was purified essentially as described [18] with some modifications. BL21(DE3)A cells transformed with pLS252 were obtained from ATCC. Following a 5 hour expression, cells were harvested, resuspended in 20 mM Tris (pH 7.6), 0.5 M NaCl, 0.1 mM EDTA, 1 mM BME and lysed. Following centrifugation, nucleic acids were removed by polyethyleneimine (PEI) treatment. The PEI supernatant was treated with 75% ammonium sulfate and subjected to centrifugation. The pellet was resuspended in 20 mM Tris pH 7.6, 100 mM NaCl, 0.1 mM EDTA, 1 mM BME and dialyzed against Buffer A (20 mM Tris pH 7.6, 150 mM NaCl, 0.1 mM EDTA, 5 mM BME). The dialysate was loaded onto a phosphocellulose column and eluted with buffer B (20 mM Tris pH 7.6, 1 M NaCl, 0.1 mM EDTA, 5 mM BME). Fractions containing DpnI were pooled, dialyzed against buffer A and loaded onto an EMD sulfate column. Fractions containing DpnI were again pooled, dialyzed against buffer A and loaded onto an EMD sulfate column to remove any remaining contaminates.

DpnI was biotin labeled with the EZ-Link Sulf-NHS-biotin kit (Pierce, Rockford, IL) following the manufacturer's protocol. The extent of biotinylation was evaluated using the HABA assay (Pierce). Each mole of DpnI was found to contain 4-5 mole of biotin.

### Restriction activity assay

1 µg of pUC19 was digested in the presence of 100 ng of purified DpnI, DpnI-biotin or with 20 U of commercial DpnI in 20 mM Tris-HCl (pH 7.6), 50 nM NaCl, 10 mM CaCl_2_, with or without 20 mM MgCl_2_ for 1 hour at 37°C. Reactions were stopped by the addition of loading buffer containing SYBR green (Life Technologies, Carlsbad, CA). DNA was separated on a 1.5% TBE agarose gel.

### Generation of template DNA

DNA was PCR amplified from pUC19 using primers (IDT, San Diego, CA) that resulted in a 477 nt fragment (Forward- TCTGCGCTCTGCTGAAGCCAGTTAC; reverse- GCTGATAAATCTGGAGCCGGTGAGC) or a 651 nt fragment (forward- GGCAGCAGCCACTGGTAACAGGATT; reverse- GATGGAGGCGGATAAAGTTGCAGGA). The 477 nt fragment was treated with *dam* methyltransferase (NEB) resulting in DNA containing the G^m6^ATC modification. All fragments were gel-purified using agarose gel electrophoresis and the MinElute Gel Extraction kit (Qiagen, Venlo, Limburg).

### Electrophoretic mobility shift assay

EMSA was carried out as previously described [19] with some modifications. FAM-labeled duplex oligonucleotide containing one G^m6^ATC site with the top strand sequence FAM-GCAGG^m6^ATCAACAGTCACACT (TriLink, San Diego, CA) was incubated with DpnI (or DpnI-biotin) in the presence of 20 mM Tris-HCl, 50 mM NaCl, 10 mM CaCl_2_, 1 mg/ml BSA and 10 µg/ml salmon sperm DNA for 30 minutes at room temperature. Glycerol was added to a final concentration of 10% and the samples loaded onto a 20% TBE acrylamide gel (Life Technologies) that had been pre-run for 2 hours at 4°C with TBE. Samples were subjected to separation at 200 V for 1.75 hours. FAM-labeled DNA was imaged using an AlphaImager (Protein Simple, Santa Clara, CA).

### DpnI pull-down assay

#### Preparation of DpnI-coated magnetic beads

20 µl streptavidin magnetic beads (NEB) were washed twice with Binding Buffer (10 mM Tris pH 7.9, 50 mM NaCl, 10 mM CaCl_2_, 0.01% Tween 20). Biotinylated DpnI was added to the beads at 10 ng DpnI/µl beads. After mixing by pipetting, the beads were washed twice with Binding Buffer and used for binding reactions.

#### DNA pull-down

DNA samples were prepared in Binding Buffer. The assay was performed either in 1.7 ml microcentrifuge tubes or in a 96-well plate. 50 µl DNA samples were added to the DpnI coated beads. The beads were mixed by end-over-end rotation or on a plate shaker for 5 minutes to 1 hour. Magnetic beads were separated using either a tube magnetic stand (Life Technologies) or a plate magnet (Millipore, Billerica, MA). The beads were washed once with Wash Buffer (10 mM Tris pH 7.9, 500 mM NaCl, 10 mM CaCl_2_, 0.1% Tween 20) followed by one Binding Buffer wash. Beads were resuspended in 50 µl of Binding Buffer for qPCR analysis.

For gel analysis and next-generation library preparation, the DNA was eluted from beads by incubation with 50 µl 5 M guanidinium thiocyanate at room temperature for 5 minutes. The eluent was transferred to a 3500 MWCO dialysis tube (Thermo Scientific, Waltham, MA) and dialyzed against distilled water for 1 hour at room temperature.

### Genomic DNA qPCR analysis

Primers were synthesized by IDT and probes were made by Life Technologies. Reactions were prepared using the QuantiProbe FAST PCR Kit (Qiagen) except for the DYZ assay which was prepared with TaqMan Universal Master Mix (Life Technologies). Reactions were cycled once at 95°C for 3 minutes followed by 40 cycles of 95°C for 3 seconds and 60°C for 30 seconds on an ABI 7300. The universal bacterial 16S assay has been described previously [20]. Assays specific for Human RNaseP, human TERT and Arabidopsis ACT2 gene were obtained from Life Tech. *E. coli* 16S assay: forward -CCAGGGCTACACACGTGCTA; reverse - TCTCGCGAGGTCGCTTCT; probe - AATGGCGCATACAAA. Human DYZ assay: forward - TCGAGTGCATTCCATTCCG; reverse - ATGGAATGGCATCAAACGGAA; probe - TGGCTGTCCATTCCA. Relative abundance was calculated using either a standard curve or the delta Ct method. For the universal 16S assay, standard curves were generated using the genomic DNA of the organism being tested to correct for the varied copy number of the 16S gene.

### Preparation of synthetic mixture

Bacterial genomes were obtained through the ATCC or BEI as listed and concentrations determined using the Qubit dsDNA HS assay (Life Technologies). Bacterial genomes were diluted with water to obtain the desired concentrations (Table 1) and validated again using Qubit dsDNA HS assay before assembly of the final synthetic mix.

**Table 1:** DpnI pulls down genomic DNA from different organisms with varying efficiency.

Family	Organism	Strain	Gram	DamMT	DpnI Pull Down Efficiency*
*Aeromonadaceae*	*Aeromonas hydrophila*	ATCC 7966	-	+	++
*Enterobacteriaeceae*	*Enterobacter cloacae*	ATCC 13047	*-*	+	++
	*Escherichia coli*	K12	*-*	+	++
	*Klebsiella pneumoniae*	ATCC 700721	*-*	+	++
	*Proteus mirabilis*	ATCC 12453	*-*	+	++
	*Salmonella typhimurium*	SU453	*-*	+	++
	*Serratia marcescens subsp. marcescens*	ATCC 13880	*-*	+	++
	*Yersinia pestis*	China CDC	*-*	+	++
	*Yersinia pseudotuberculosis*	ATCC 13979	*-*	+	++
*Pasterellaceae*	*Haemophilus influenzae*	ATCC 51907	*-*	+	++
	*Haemophilus parahaemolyticus*	ATCC 10014	*-*	+	++
	*Haemophilus parainfluenzae*	ATCC 33392	*-*	+	++
*Legionellaceae*	*Legionella pneumophila*	ATCC 33152	-	(+)	++
*Campylobacteraceae*	*Campylobacter jejuni subsp. jejuni*	ATCC 700819	*-*	(+)	+
*Helicobacteraceae*	*Helicobacter pylori*	ATCC 700824, J99	*-*	(-)	+
*Burkholderiaceae*	*Burkholderia mallei*	CRP 23344	-	(-)	+
	*Burkholderia cepacia*	CRP BRUK102	-	-	+
*Brucellaceae*	*Brucella abortus*	CRP 2308	-	-	+
*Pseudomonadaceae*	*Pseudomonas aeruginosa*	ATCC 47085	-	-	+
*Bacillaceae*	*Bacillus anthracis*	Sterne	+	-	-
*Enterococcaceae*	*Enterococcus faecium*	ATCC 51559	+	-	-
*Eukaryota - Fungi*	*Aspergillus fumigatus*	MYA-4609	N/A	-	+/-
*Eukaryota - Brassicaceae*	*Arabidopsis thaliana*		N/A	-	-
*Eukaryota - Hominidae*	*Homo sapiens*, male		N/A	-	-

*Recovery as compared to input by qPCR.

-Less than 2%, +/-2–10%, +10–50%, ++50–100%.

### DNA isolation from saliva

The PowerSoil DNA isolation kit (MO BIO Laboratories, Carlsbad, CA) was used to extract DNA from 1 ml of pooled human saliva (BioReclamation, Farmingdale, NY). The DNA was eluted in DpnI Binding Buffer and 400 ng of the DNA was subjected to the DpnI pull-down assay. The input, unbound, and bound/eluted fractions were used to prepare sequencing libraries.

### DNA isolation from creek water

A 1000 ml water sample was collected from a creek 25 meters downstream from a sedimentation pond used for primary passive treatment of ground water run-off. A 100 ml aliquot was filtered over a 0.2 µm Nalgene sterile analytical filter unit (Thermo Scientific) prior to DNA extraction with the PowerWater DNA Isolation Kit (MO BIO Laboratories). A 150 ng aliquot of the DNA was subjected to the DpnI pull-down assay. The input, unbound, and bound/eluted fractions were used to prepare sequencing libraries.

### Library preparation and sequencing

The Nextera DNA Sample Preparation Kit (Illumina, San Diego, CA) was used to prepare libraries from input, unbound, and bound/eluted fractions from DpnI pull-down assays. Manufacturer's instructions were followed for the library preparation except for recommended number of PCR cycles, which were varied according to the amount of DNA. For the synthetic mixture, they were as follows: Input – 7 cycles, DpnI bound – 10 cycles, DpnI unbound – 7 cycles. Libraries were sequenced following the manufacturer's instructions for the HiSeq 2500 Rapid Run mode to obtain 50 nucleotide read lengths. The files corresponding to all the raw reads generated in this study are publicly available at the NCBI Short Read Archive (SRP044748).

### Sequence analysis

For microbial taxa identification, Illumina data sets were analyzed by an automated pipeline (ZovaSeq from Zova Systems, LLC, San Diego CA) in which identifying sequence reads are assigned to specific microbial taxa when a given read length is found to occur uniquely within the taxa as defined by the NCBI taxonomy database [21], [22]. Relative abundance was calculated using two methods which gave equivalent results: tallying the number of ZovaSeq identifying reads for each bacterial taxa or by using Bowtie 1.0.0 to map reads to all identified organisms in the sample by perfect match. For known higher eukaryotes in the sample (*Homo sapiens, Oryza sativa*) reads were mapped using Bowtie 1.0.0 with parameters allowing 2 mismatches in a 28 bp seed region.

Relative enrichment of the DpnI bound versus input samples were determined by the following equation: 
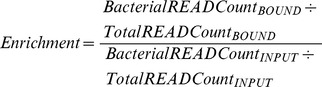
Relative enrichment as compared with human DNA was determined by dividing DpnI enrichment for the organism of interest by DpnI enrichment for human.

## Results

**Figure 1:**
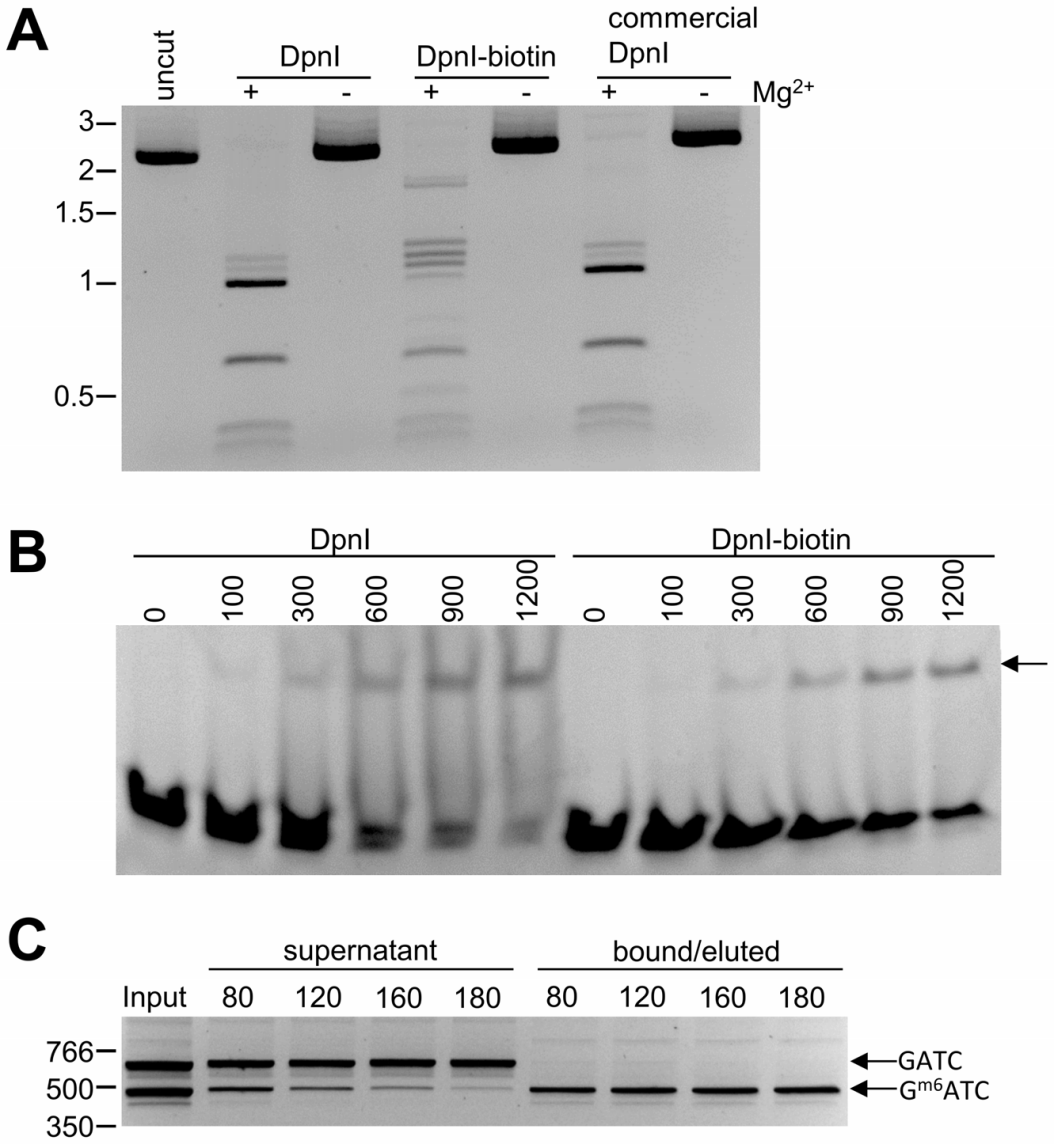
Analysis of biotinylated DpnI. (A) pUC19 was incubated with DpnI, DpnI-biotin or commercially sourced DpnI in the presence or absence of 10 mM magnesium chloride. The digested fragments were separated on a 1.5% agarose gel. (B) A FAM-labeled DNA duplex containing one G^m6^ATC site was incubated with increasing amounts of DpnI or DpnI-biotin (0 to 1200 ng). The reactions were separated on a 20% TBE gel and analyzed with fluorescence imaging. (C) An unmethylated 651 bp DNA fragment and a Dam-methylated 477 bp DNA fragment were combined and incubated with increasing amounts of immobilized DpnI-biotin (80–180 µl). DNA was eluted using GTC and desalted. All fractions were separated on a 3% agarose gel.

6mA is a frequent prokaryotic DNA modification that has only rarely been reported in eukaryotic genomes [13]. Since DpnI is one of a limited number of methyl-directed Type II restriction endonucleases that depend on the presence of 6 mA to bind and cut its target DNA sequence [16], [17], we surmised that it could effectively bind G^m6^ATC containing genomes for enrichment, allowing segregation away from non-methylated GATC DNA. To test this, we covalently bound biotin to DpnI to facilitate immobilization of the enzyme onto streptavidin coated particles. This necessitated purification of DpnI since commercial sources for the enzyme are dilute and contain other proteins that prevent us from selectively biotinylating the restriction enzyme. The activity of purified DpnI both before and after biotinylation was analyzed by restriction digestion of pUC19 isolated from DamMT+ *E. coli*. DpnI and DpnI-biotin were both found to be active when compared to commercially available enzyme, with a slight reduction in activity observed when the protein was biotinylated (Figure 1A).

To effectively bind and separate G^m6^ATC DNA fragments from a mixture, the cleavage activity of DpnI must be prevented. We tested DpnI digestion of pUC19 in the absence of magnesium ions and did not observe cleavage activity, as previously reported [16]. Since the absence of magnesium might also affect the binding of DpnI to its target, we tested both DpnI and DpnI-biotin in an electrophoretic mobility shift assay. A FAM-labeled oligonucleotide duplex containing a single G^m6^ATC sequence was incubated with increasing amounts of DpnI and DpnI-biotin. Both DpnI and DpnI-biotin are able to bind and shift G^m6^ATC containing DNA in the absence of magnesium and no noticeable decrease in the binding affinity is observed when DpnI is biotinylated (Figure 1B).

To test our hypothesis that DpnI could be used to separate G^m6^ATC containing DNA from fragments without G^m6^ATC sites, we used a mixture of a 477 bp Dam-methylated DNA fragment and a 651 bp non-methylated fragment. The two fragments both contained seven GATC sites and were derived from overlapping regions in pUC19 to minimize bias caused by sequence differences. DpnI-biotin was immobilized onto streptavidin-magnetic particles and titrated into a mixture of the two fragments. DNA that bound to the DpnI-coated particles was eluted and desalted. All fractions were separated by electrophoresis on an agarose gel. An increase in the amount of DpnI-beads resulted in further depletion of the 477 bp fragment. The eluted fractions contained only the 477 bp fragment (Figure 1C, lanes 6–9) leaving the non-methylated 651 bp fragment in the supernatant (Figure 1C, lanes 2–5). Thus immobilized DpnI specifically bound G^m6^ATC containing DNA (477 bp) which could be purified away from other fragments.

**Figure 2:**
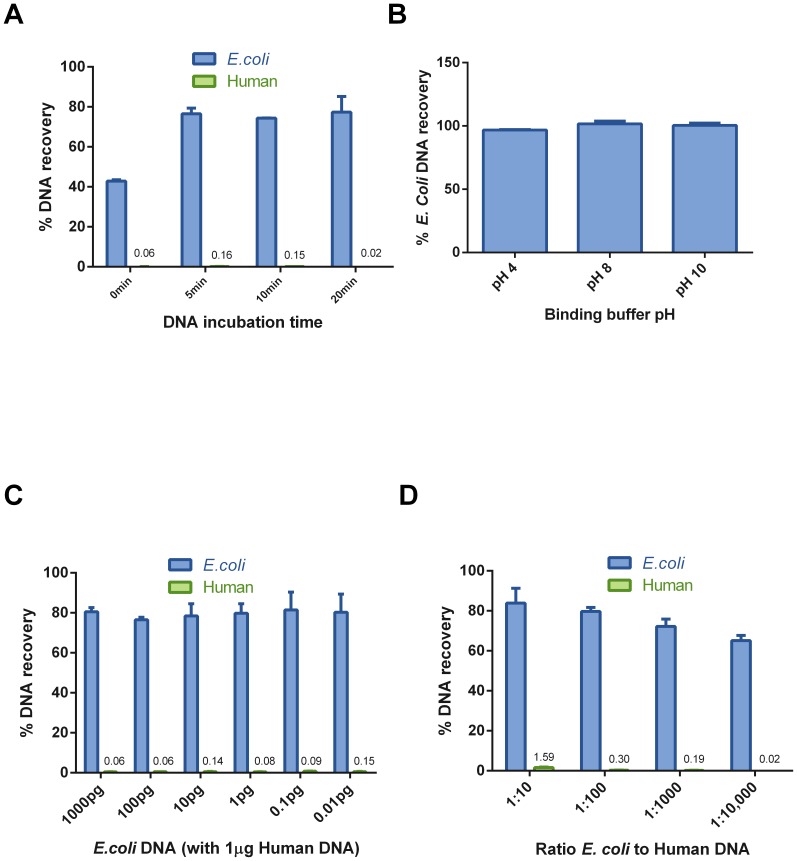
Efficiency and range of DpnI pull-down. (A) Immobilized DpnI was incubated with a mixture of *E. coli* and human DNA for varying amounts of time. 40% of *E. coli* DNA binding occurs on less than one minute. Less than 0.2% of human DNA binds to DpnI. (B) Immobilized DpnI was incubated with *E. coli* DNA in buffers with pH of 4, 8 or 10. Almost all *E.coli* DNA was recovered in the range of pH tested. (C) A fixed amount of human DNA (1 µg) was mixed with decreasing levels of *E. coli* DNA and then incubated with immobilized DpnI. Approximately 80% of *E. coli* DNA is recovered down to levels of 10 fg. All data shown is the average of three experiments. (D) A fixed amount of *E. coli* DNA (1 ng) was mixed with increasing amounts of human DNA and then incubated with immobilized DpnI. There is a slight decrease in the recovery of *E. coli* DNA with increasing amounts of human DNA. However, even when human DNA is present at 10,000x, DpnI recovers over 70% of *E. coli* DNA.

After observing efficient segregation of specific G^m6^ATC DNA fragments, we investigated whether DpnI-biotin was suitable for isolating a G^m6^ATC-containing genome when mixed with GATC-containing genomes. A synthetic mix containing 1 ng *E. coli* and 500 ng of Human genomic DNA was prepared and incubated with immobilized DpnI. After separation, fractions were analyzed using qPCR. We found that DpnI-coated particles isolated *E. coli* genomic DNA with high efficiency (Figure 2A), binding nearly 80% in 5 minutes. Enrichment was also specific, with 99.6% of Human DNA remaining unbound. Comparable isolation efficiency was observed for the DNA mixtures prepared in buffers ranging from pH 4 to 10 (Figure 2B). Additionally when fragment sizes were at least 3 kb, DpnI binding was not significantly affected, but did decrease with smaller fragments (Figure S3).

The relative genomic composition of complex samples varies widely. We therefore tested the limits of DpnI separation by incubating various amounts of *E. coli* and human DNA with immobilized DpnI. To test the sensitivity of DpnI separation, the level of human DNA was held constant at 1 µg and *E. coli* DNA was titrated from 1 ng to 10 fg. We observed approximately 80% recovery of *E. coli* DNA and rejection of 99.5% of human DNA. Sensitivity was observed to 10 fg *E. coli* DNA, the detection limit of the qPCR assay used (Figure 2C). This demonstrates efficient separation by DpnI of G^m6^ATC containing DNA when present at as low as 10^−8^ of the level of eukaryotic DNA.

We next tested the ability of DpnI to exclude human DNA present at high concentrations. When the concentration of *E. coli* DNA was held constant at 1 ng while increasing the concentration of human DNA, we observed *E. coli* DNA recovery as high as 82% and exceeding 60% even in the presence of 10 µg of human DNA, a 10,000-fold difference (Figure 2D). These results demonstrate that DpnI DNA segregation is effective and efficient with differing ratios of target versus non-target DNA.

We next examined how efficiently DpnI binds genomes from a variety of organisms including some that are clinically relevant [23]. For each organism of interest, 1 ng of bacterial genomic DNA was combined with 1 µg of human DNA. DNA mixtures were incubated with immobilized DpnI. Following segregation, DNA in the DpnI bound and unbound fractions were analyzed by qPCR. DpnI successfully bound and separated genomic DNA from gram-negative organisms known to express DamMT (Table 1). The range of recovery was between 50% and 100% of the measured input. For gram-negative bacteria not known to have a DamMT gene, recovery was lower, from 10% to 45% of the measured input, but still significantly higher than binding to human DNA. Binding of gram-positive bacterial DNA was less than 3% and binding to eukaryotic DNA was below 0.5%. We conclude that DpnI can be used to efficiently bind and segregate genomes from a wide variety of organisms with very little binding to eukaryotic DNA.

**Figure 3:**
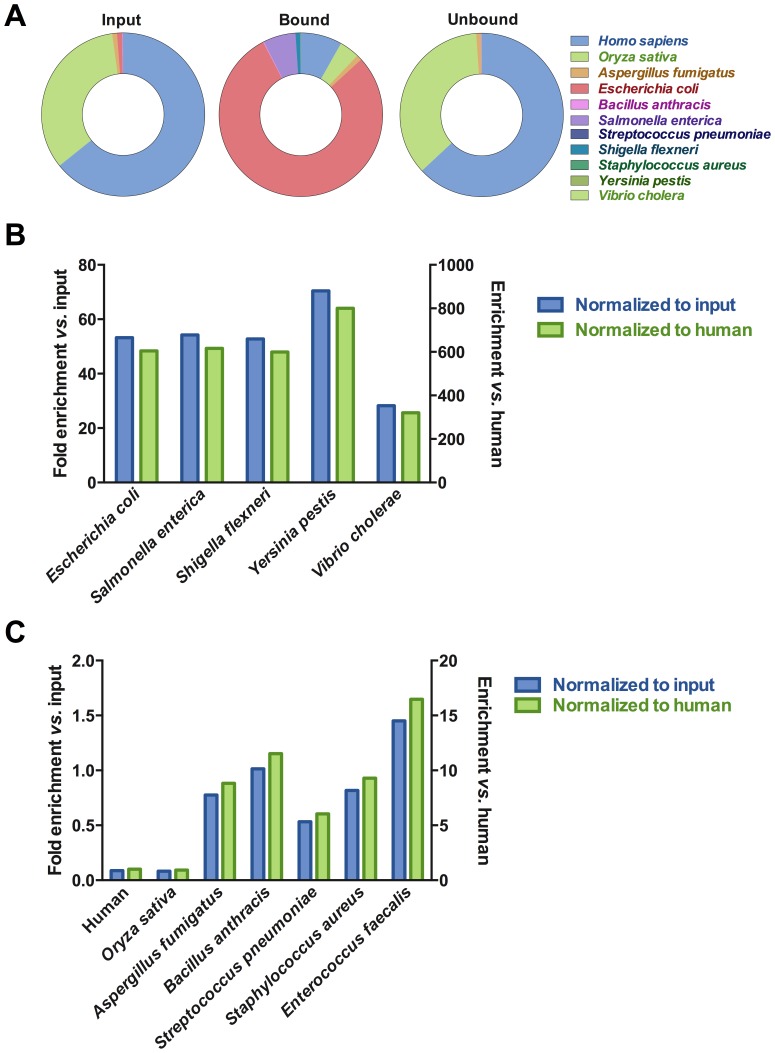
DpnI enriches prokaryotic DNA as determined by NGS. (A) NGS reads from the input, bound and unbound fractions of the synthetic mix. Reads from the input map overwhelmingly to human and rice, with less than 10% mapping to prokaryotes in the synthetic mixture. Less than 10% of the reads from the bound fraction map to human with the majority mapping to *E. coli*. (B) DamMT+ genomes are enriched 30 to 70-fold versus their input levels, and 300 to 800-fold versus human DNA. (C) Genomes that lack DamMT are enriched when compared to human and rice.

To test how well DpnI enrichment can improve the coverage and read depth of prokaryotic DNA in a mixture, we designed a synthetic mixture of genomic DNA that included both eukaryotic and prokaryotic DNA (Table 2). Human DNA made up the bulk of the mixture at over 97% by weight. DNA from rice (1%) and *Aspergillus* (1%) was added to represent plant and fungal genomes, respectively. Microbe genomes were added in a pair-wise fashion. Each pair consisted of an equal amount of DNA from a DamMT+ and a DamMT- organism, and subsequent pairs were diluted ten-fold to test the limit of DpnI enrichment. The DNA mixture was subjected to DpnI segregation. The DNA from the bulk mixture, the unbound fraction and the bound/eluted fraction were used to prepare sequencing libraries. We found that the number of reads from eukaryotes was dramatically reduced in the DpnI-bound fraction (Figure S5). Reads mapping to the human genome made up 59% of the mapped reads in the synthetic mix input but only 5% in the DpnI-bound fraction. The reads mapping to *Oryza* (rice) were also greatly reduced, from 31% of the mapped reads in the input sample to 2.5% of the mapped reads in the bound fraction (Figure 3A).

**Table 2:** Genome mix used for sequencing and relative enrichment results.

Organism in Input Mixture				Relative Enrichment	
Species	Strain	Genome Size	% by mass	Bound vs. Input	Organism vs. Human
*Homo sapiens*		3,209,290,000	96.80%	0.09	N/A
*Oryza sativa*		382,780,000	1.00%	0.08	0.9
*Aspergillus fumigatus*	Af293	29,390,000	1.00%	0.8	9.1
*Escherichia coli*	O157:H7 str. EDL933	5,620,000	1.00%	57	654
*Bacillus anthracis*	Sterne	5,228,663	0.10%	1.2	13.5
*Salmonella enterica*	Ty2	4,790,000	0.10%	58	666.1
*Streptococcus pneumoniae*	R6	2,038,615	0.01%	0.7	7.8
*Shigella flexneri*	2457T	4,600,000	0.01%	58.9	676.2
*Staphylococcus aureus*	Mu50	2,903,147	0.001%	0.9	9.8
*Yersinia pestis*	A1122	4,660,000	0.001%	72.1	827.2
*Enterococcus faecalis*	V583	3,360,000	0.0001%	ND*	ND*
*Vibrio cholera*	N16961	3,745,000	0.0001%	75.4	865.2
*Pantoea ananatis*		N/A**	N/A**	55.9	641.7

ND: Not determined. N/A: Not applicable.

*E. faecalis was not detectable in the Input fraction.

**P. ananatis was not knowingly added in the sample mix but is a probable contaminant of the rice genome (*O. sativa*).

**Figure 4:**
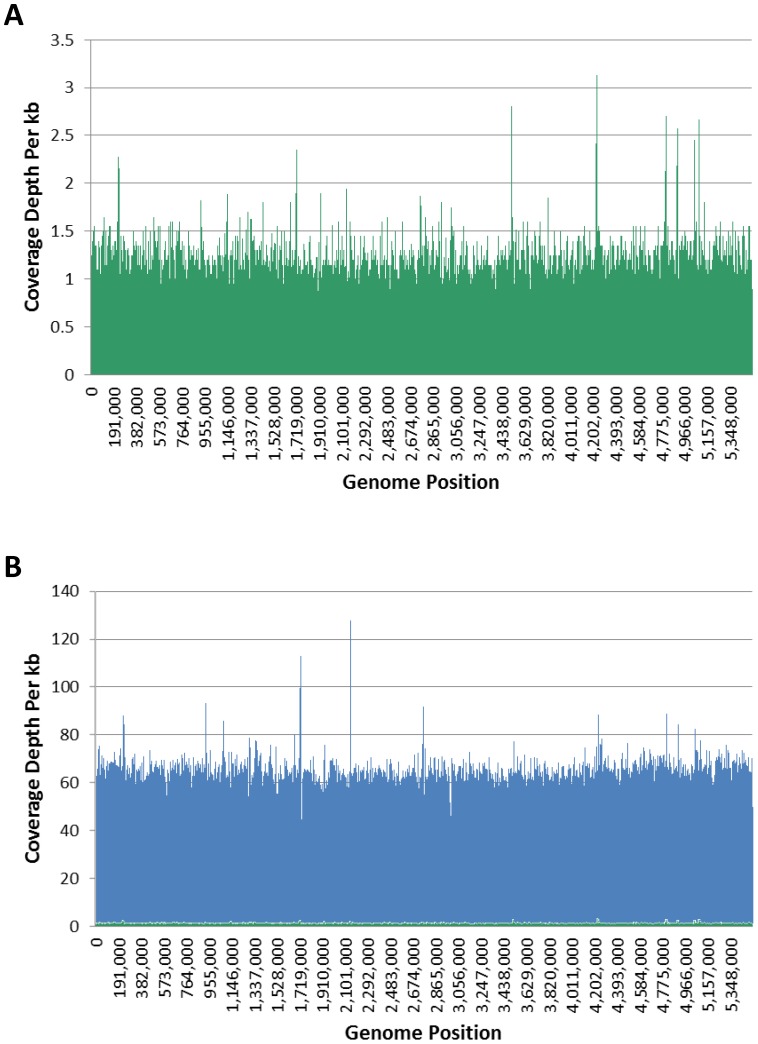
NGS coverage maps for *E. coli* from input (A) and bound (B) fractions of the synthetic mixture. Reads were mapped to *E. coli* O157:H7 EDL933 and binned into 1000 bp bins. (A) The average depth of coverage is 0.5 for *E. coli* in the input fraction (green), with 62% of the genome covered. (B) For the bound fraction (blue), the average depth of coverage increases to 60 and 99.5% of the genome is covered. The input fraction (green) is also plotted here for comparison to the bound fraction at the same scale.

Surprisingly, we observed that DNAs from all microbial organisms, not just from DamMT+ bacteria, were enriched compared to human and rice (Figure 3B, 3C and Figure S5). DNA from DamMT+ bacteria was most effectively enriched, up to 70-fold compared to input levels and up to 800-fold when directly compared to human (Figure 3B). The *E. coli* DNA in the mixture was enriched from comprising less than 1% of the reads in the sample input to over 50% of the reads in the bound fraction. This resulted in improved sequencing coverage of the *E. coli* genome. Only 67% of the *E. coli* genome sequence was covered by reads in the input sample. Following DpnI enrichment, >99% of the *E. coli* genome sequence was covered, with a depth of coverage averaging 40 reads. Furthermore, there was no discernable coverage bias in the enriched genomes (Figure 4B), indicating that DpnI enrichment can be used to greatly improve whole genome sequencing. A similar pattern of enrichment was observed for the remaining DamMT+ organisms.

**Figure 5:**
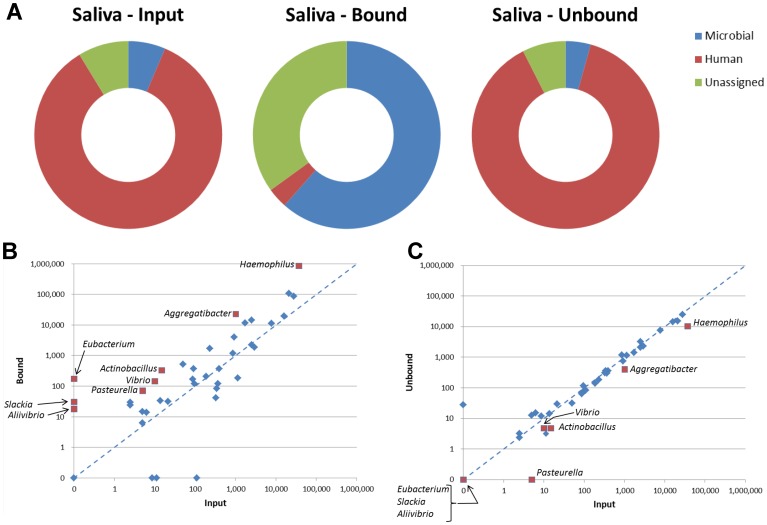
NGS data of saliva samples. (A) Donut plots depicting relative abundance of identifying reads for microbial and human genomes in input, bound and unbound samples. (B, C) Pairwise plots of sample fractions versus input. (B) Bound vs. input. (C) Unbound vs. input. Plotted points are identifying reads of genera. To facilitate direct visual comparison between samples reads were normalized to 10 M total.

As an exemplar clinical sample, DNA in saliva is overwhelmingly derived from human cells [24], with prokaryotic DNA making up less than 4%. We isolated DNA from saliva and performed a DpnI separation. The input, bound/eluted and unbound fractions were sequenced. Whereas human reads made up over 75% of the total reads in the input sample, following DpnI enrichment less than 5% of the total reads were human (Figure 5A). Prokaryotic reads increased from less than 5% of the total reads to over 50% in the DpnI-enriched fraction. There are a significant number of reads that could not be assigned to any organism. This is likely due to the high number of unsequenced organisms in the sample. The most abundant genera in the sample were *Haemophilus, Neisseria, Veillonella, Prevotella* and *Streptococcus*. Together these five genera comprised 87% of reads mapped to prokaryotes. As expected, a subset of the organisms was highly enriched in the bound fraction while some organisms were not enriched and yet another set were depleted (Figure 5B). *Haemophilus, Aggregatibacter, Actinobacillus, Vibrio* and *Treponema* were all enriched ten-fold in the bound fraction compared to input (Figure 5B). *Haemophilus parainfluenzae* was a major component of both the input and bound fractions and was enriched 36-fold compared to input. Though not enriched, *Prevotella*, an organism closely associated with dental carries [25], is still a major component of the bound fraction. Other organisms were undetectable in the input fraction but had mapped reads in the bound fraction (Figure 5B and Table S1).

**Figure 6:**
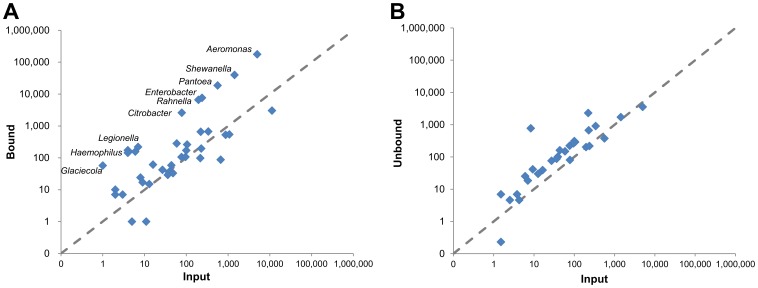
NGS data of creek samples. Pairwise plots of sample fractions versus input. (A) Bound vs. input. (B) Unbound vs. input. Plotted points are identifying reads of genera arbitrarily normalized to 10 M reads total.

We next isolated DNA from a water sample collected from a creek after a heavy rain and subjected it to segregation by DpnI. The identified genera segregated into three distinct groups in the bound fraction: highly enriched, slightly enriched and non-enriched (Figure 6A). Eleven genera were enriched over 20-fold compared to input. Of these, *Aeromonas, Shewanella, Pantoea, Enterobacter* and *Rahnella* were the most abundant in the bound fraction. For example, we found a high number of identifying reads in the bound fraction that mapped to the fish pathogen *Aeromonas salmonicida* (over 18% of mapped reads and 0.48% of the total reads). The same organism represented less than 6% of mapped reads and 0.014% of the total reads in the input (Figure S1). The coverage we observed suggests that the sequenced organism is a close relative of *Aeromonas salmonicida*. DpnI segregation resulted in nearly 35-fold enrichment of this organism's DNA.

Having succeeded in efficiently segregating DNA genomes with DpnI, we investigated whether this approach might be applicable to other restriction enzymes. DpnII is known to have the opposite activity of DpnI in that it recognizes and cuts only non-methylated GATC sequences and DpnII activity is blocked by 6 mA. We therefore expected DpnII to bind to human, but not *E. coli* genomic DNA. Similar to our experiments with DpnI, we immobilized DpnII to test its ability to separate a mixture of 1 ng of human DNA and 500 ng of *E. coli* DNA. DpnII was able to enrich the human DNA with minimal binding to *E. coli* DNA (Figure S2). Therefore restriction endonuclease-mediated DNA separation is not limited to DpnI.

## Discussion

Type II restriction endonucleases have been selected during evolution to ensure they do not cut their own DNA, a suicidal event, while quickly binding to and digesting any foreign DNA that lacks the correct methylation pattern [26]. We demonstrate that manipulation of *in vitro* conditions enables DpnI to bind but not cut DNA containing its target sequence. While the binding affinity of DpnI has not been determined, several restriction enzymes have been measured in the picomolar [27], [28] to nanomolar range [29], [30] and our results support the use of restriction enzymes as strong and specific DNA binding proteins.

DpnI binding to target DNA was rapid, with 75% of *E. coli* DNA bound after only 5 minutes (Figure 2A). We also observed highly specific binding with over 99.5% of human DNA excluded and over 80% of targeted *E. coli* DNA binding (Figure 2A, C and D). This rapid and exquisite target discrimination by DpnI *in vitro* is a reflection of the natural ability of restriction endonucleases to quickly scan and locate target sequences in large amounts of DNA *in vivo* [31]. Immobilized DpnI can be used to differentially bind and segregate prokaryotic DNA present at 1/10,000 the level of eukaryotic DNA (Figure 2D). Efficient removal of background human genetic material enables pathogen DNA to be concentrated to achieve sensitive detection which could be particularly useful for un-culturable pathogenic bacteria. This feature could be exploited for the diagnostic detection of trace amount of pathogens in clinical samples such as blood from patients with septicemia, a serious infection that lacks an early detection method [32].

One critique of using a methyl-directed binding protein to enrich DNA is that the process may introduce coverage bias with more reads observed in close proximity to the protein binding site. However, when samples were separated by DpnI and then analyzed by NGS, DpnI enrichment resulted in very low sequence coverage biases (Figure 4). The even coverage is likely due to the frequency and distribution of DpnI binding sites in target DNA. For example, in *E. coli* O157:H7, there are approximately 42,000 GATC sites, 94% of which have been shown by SMRT sequencing to be adenine methylated with an average gap between GATC sites of about 250 bp [12]. Additionally, DpnI segregation generated low biases when input DNA fragments were above 3 kb (Figure S3). Thus typical DNA isolation procedures are sufficient to achieve efficient DpnI segregation. Biases could arise however if smaller bacterial fragments, from degraded DNA for instance, are present.

We predicted little or no binding to eukaryotic DNA and highly specific binding to DNA from DamMT+ bacteria. We did not anticipate the low level binding of DpnI to micro-organisms not known to contain G^m6^ATC sites (Figure 3B). This *in vitro* non-canonical binding may simply reflect a difference in DNA binding affinity compared to the more rigorously studied specificity of restriction activity. Published factors known to affect restriction specificity of DpnI include the presence of non-GATC sequences that contain a methylated adenine residue [33] and DNA topology effects [34]. Alternatively, DNA modifications other than 6mA may be affecting DpnI binding specificity. Although DpnI needs a G^m6^ATC site to cut, it appears that at least some amount of binding occurs when that pattern is absent and that binding decreases in the presence of CpG methylation. We observed that when the *Aspergillus fumigatus* genome which is not known to contain G^m6^ATC is treated with a CpG methyltransferase, binding drops significantly (Figure S4). It is unknown whether this is a differential feature of binding versus digestion or an artifact of biotinylation. A more in-depth study of DpnI binding patterns is needed to better understand the binding to DNA from DamMT- organisms.

Observations to date suggest that methyl signatures created by restriction modification systems are only sporadically distributed amongst microbial taxa [26], [35]. In contrast, orphan MTases, such as DamMT, are often conserved across extensive groups of bacteria which rely on these methylation patterns to control crucial cellular processes like chromosome replication [14], [36]. We consider DamMT+ bacteria to be part of a more expansive methylome which would include organisms which methylate at GATC sites in other contexts (*e.g*. *B. amyloliquefaciens*, BamHI GG^6m^ATCC). The broad and deep genomic coverage consistently observed when sequencing DpnI enriched DamMT+ bacterial DNA (Figure 4) suggests that the binding kinetics are equivalent across these organisms. We hypothesize that with regard to G^6m^ATC, organisms may divide into genomes that (A) have a DamMT-like density of G^6m^ATC sites and are highly enriched, (B) lower site density that are only slightly enriched and (C) those genomes with no G^6m^ATC sites. This last category may be greatly discriminated against if it possess mCpG sites, as does human DNA, or may result in an equal in abundance in the bound fraction and the input sample when CpG sites are absent (Figure 6A), as is the case for most bacteria.

We demonstrated that by purifying DNA by methylome, enrichment exceeding 50-fold of specific genomes is possible. In the case of the water sample, an organism closely related to *Aeromonas salmonicida* was highly enriched, with hundreds of thousands of non-normalized reads in the DpnI bound fraction compared to approximately 5000 in the input library. Typically, the high complexity of a microbiome would make reassembling genomes of unknown species challenging. Existing methods rely on bioinformatics, using alignment to reference genomes, nucleotide composition [37], differential coverage binning [38], or variations in gene count [9] to achieve partial assemblies. Our enrichment approach increases coverage, facilitates informatics processes and provides opportunities to characterize previously unsequenced and unculturable microbial taxa in diverse microbial communities.

Enrichment upstream of NGS allows for better coverage and increased certainty of the presence of organisms. This may be useful for samples with a very high load of eukaryotic DNA, such as those from the throat, buccal mucosa, or saliva [24]. The DpnI enrichment of pathogen DNA from saliva has several potential applications. Bacterial populations in saliva change in response to many disease conditions [39]. Identification and quantification of bacterial profiles may be important for detection of oral and/or systemic disease. With only about 100 cultivable strains out of the over 700 oral microbiota taxa [39], DpnI enrichment may provide a reliable way to identify novel bacterial species present in saliva using NGS. For example, *Aggregatibacter actinomycetemcomitans*, a strain known to be involved in periodontitis [40] was enriched 27-fold over input (Table S1). *Treponema denticola*, another strain implicated in periodontitis [40], was undetectable in the input fraction but had over 300 associated reads in the bound fraction (Table S1).

DpnI is unique in that it is a methyl-directed type II enzyme that can be used as a tool to bind DNA of a broad clade of widely studied bacteria with impacts on human health. Our demonstration that DpnII, a methyl *inhibited* type II endonuclease can also be used for differential selection of DNA opens the door to using alternative enzymes for DNA segregation. Over 300 restriction endonucleases with methyl-specific recognition specificities have been catalogued [41] potentially offering many more opportunities to discriminate genomes based on methylation patterns. By choosing restriction endonucleases with different methylation specificities, we envision the ability to stratify complex genomic mixtures into various methylomes, thus simplifying the experimental characterization of any microbiome.

The discovery of restriction endonucleases enabled the biotech revolution. These enzymes now offer a new technical utility, expanding on their natural role as discriminators of their own genomes to allow isolation of genomes from unculturable bacterial genomes present at low levels from diverse hosts and environments. Careful consideration of 6 mA, 4mC and 5mC directed or blocked endonucleases has led us to use these molecular biological tools in new ways and to develop new methodologies that promise additional insights into the natural and pathogenic microbiomes of our world.

## Supporting Information

Figure S1 **NGS coverage maps for** ***Aeromonas salmonicida*** **.** NGS reads from the creek input (orange) and bound (blue) fractions were mapped to *Aeromonas salmonicida*, grouped into 1000 nt bins and plotted.
(TIF)

Figure S2 **DpnII efficiently binds to human DNA and excludes** ***E. coli*** **DNA.** (A) Equal amounts of human and *E. coli* DNA were combined and separated with immobilized DpnII. The majority of human DNA bound to immobilized DpnII while *E. coli* DNA was left behind. (B) An excess of *E. coli* DNA (500 ng) was combined with 1 ng of human DNA then separated with immobilized DpnII. Almost 80% of human DNA bound to DpnII while nearly all of the *E. coli* DNA remained unbound. All data shown is the average of three experiments.
(TIF)

Figure S3 **DpnI binding to sheared DNA.** *E. coli* genomic DNA was sheared by sonication and the size confirmed by gel electrophoresis. Sheared and intact (genomic) DNA was subjected to DpnI separation and binding was assessed by qPCR.
(TIF)

Figure S4 **DpnI segregation of** ***Aspergillus fumigatus*** **genomic DNA.** *Aspergillus* DNA was subjected to DpnI segregation and the fractions analyzed by qPCR. When DNA was treated with M.SssI, a CpG specific methyltransferase, the amount of *Aspergillus* DNA recovered by DpnI decreased from 2% to 0.7% compared to input levels. Data shown is the average of 4 experiments.
(TIF)

Figure S5 **Pairwise plots showing reads mapped to synthetic genomic mix input DNAs and normalized to total reads for DpnI Bound versus Input fractions.** There are three methylomes represented: those with G^m6^ATC are highly enriched (above line); those that are present at the same levels of input (on line) and those that are excluded (below line).
(TIF)

Table S1 **DpnI enriches saliva organisms that are known to be involved in oral diseases.** DNA isolated from saliva was segregated with DpnI and the DNA used to prepare NGS libraries. The fold of enrichment was calculated based on NGS mapped reads normalized to total reads in the DpnI bound versus input fractions. In the cases where there were no mapped reads in the input fraction (*T. denticola*) the normalized mapped read counts are listed.
(DOC)
