# Supplementary figures and images for: Selective Microbial Genomic DNA Isolation Using Restriction Endonucleases
Source: PLoS One. 2014 Oct 3;9(10):e109061. doi: 10.1371/journal.pone.0109061 (PMC4184833; doi:10.1371/journal.pone.0109061)

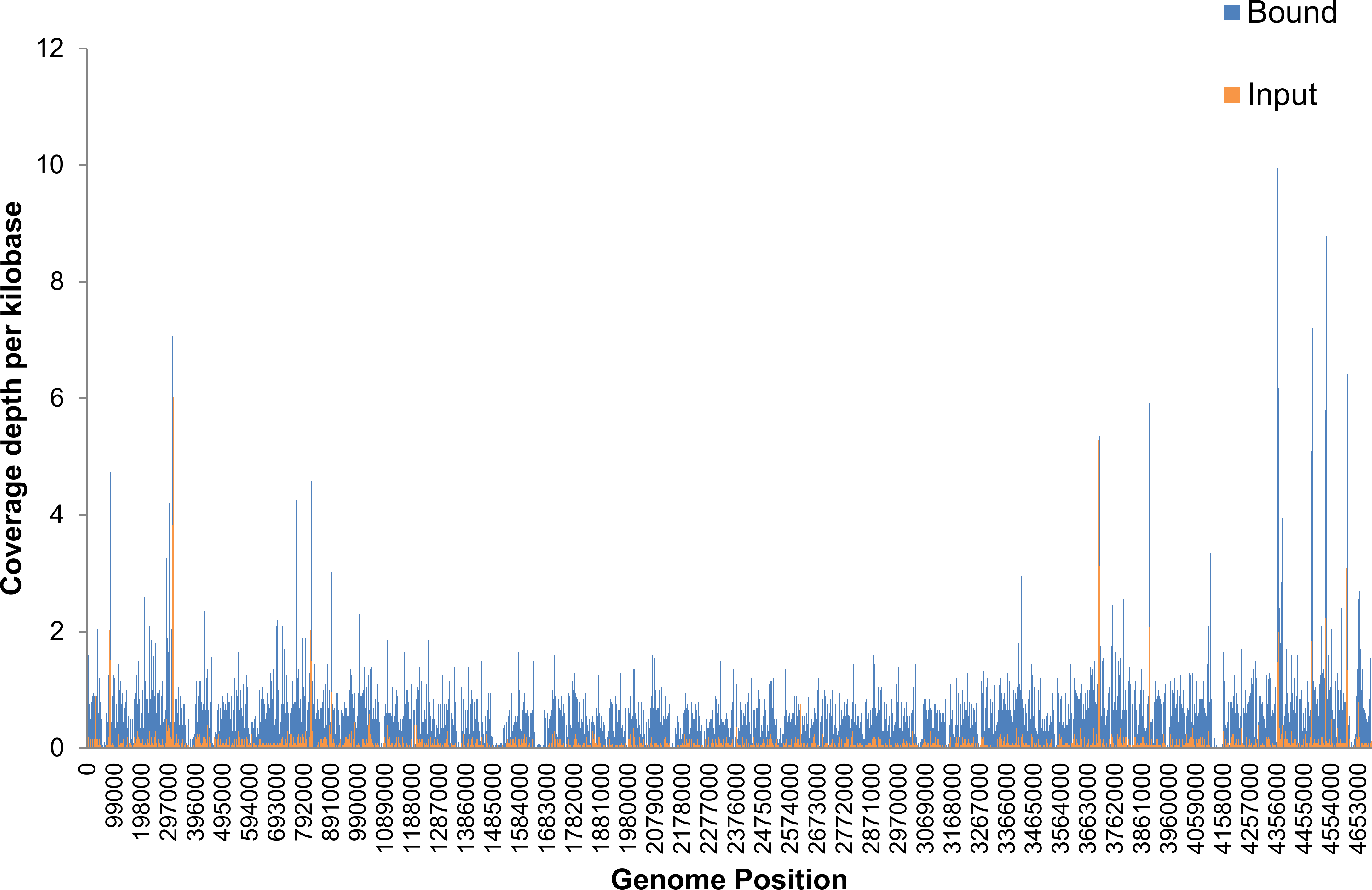

Supplement: Figure S1 — NGS coverage maps for Aeromonas salmonicida . NGS reads from the creek input (orange) and bound (blue) fractions were mapped to Aeromonas salmonicida, grouped into 1000 nt bins and plotted. (TIF) [file pone.0109061.s001.tif]

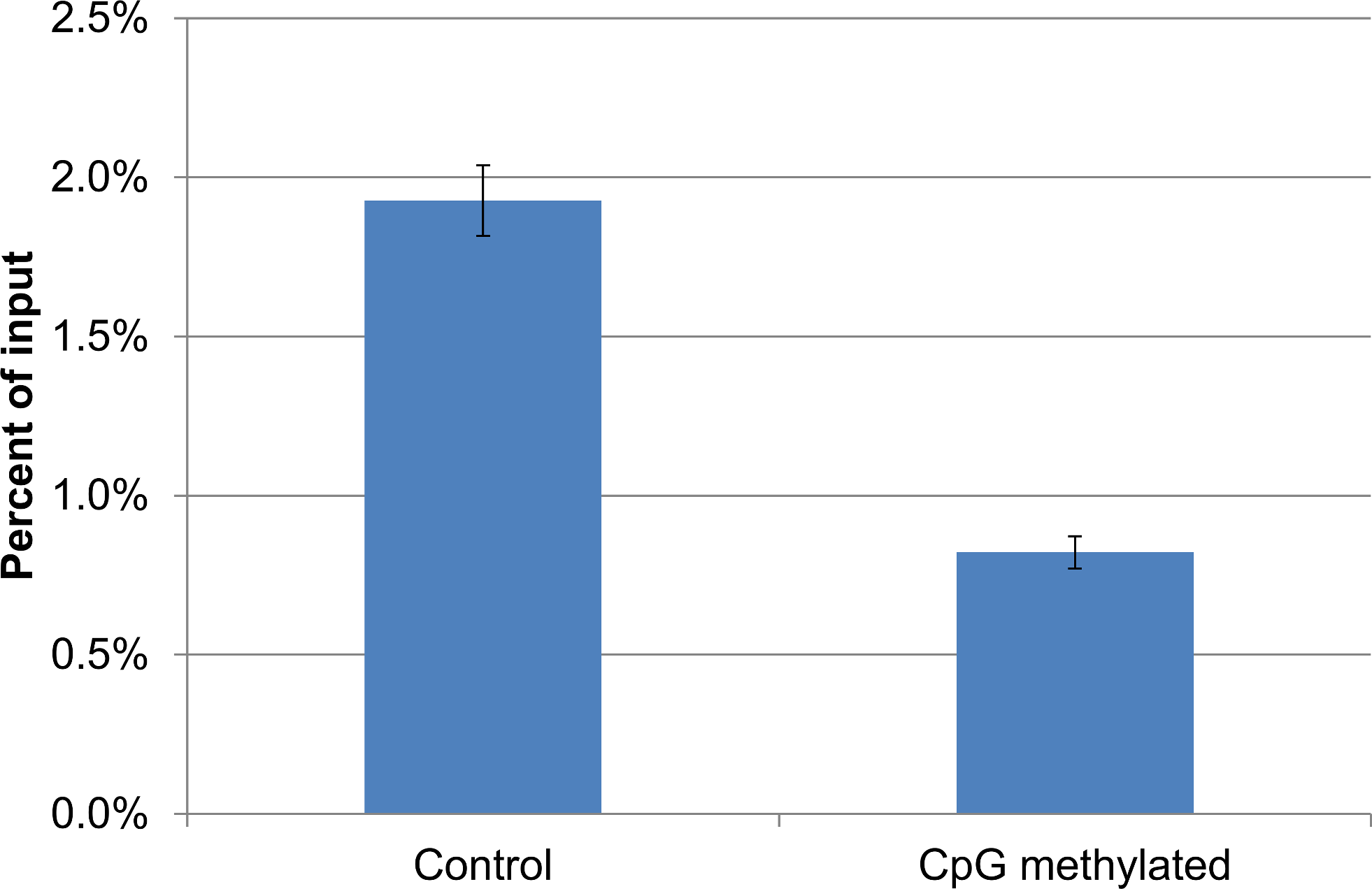

Supplement: Figure S4 — DpnI segregation of Aspergillus fumigatus genomic DNA. Aspergillus DNA was subjected to DpnI segregation and the fractions analyzed by qPCR. When DNA was treated with M.SssI, a CpG specific methyltransferase, the amount of Aspergillus DNA recovered by DpnI decreased from 2% to 0.7% compared to input levels. Data shown is the average of 4 experiments. (TIF) [file pone.0109061.s004.tif]

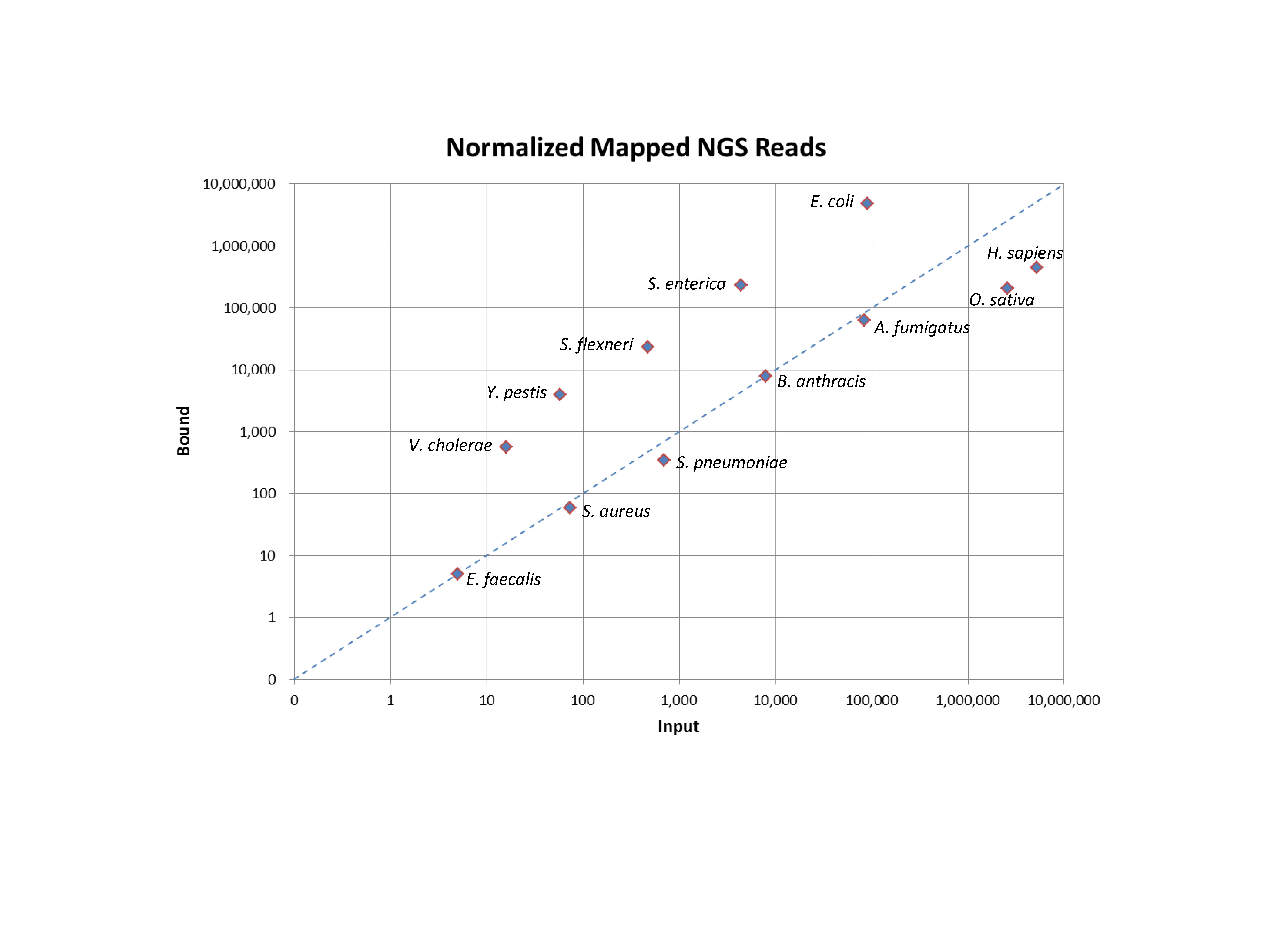

Supplement: Figure S5 — Pairwise plots showing reads mapped to synthetic genomic mix input DNAs and normalized to total reads for DpnI Bound versus Input fractions. There are three methylomes represented: those with Gm6ATC are highly enriched (above line); those that are present at the same levels of input (on line) and those that are excluded (below line). (TIF) [file pone.0109061.s005.tif]
