# Supplementary material for: Selective Microbial Genomic DNA Isolation Using Restriction Endonucleases
Source: PLoS One. 2014 Oct 3;9(10):e109061. doi: 10.1371/journal.pone.0109061 (PMC4184833; doi:10.1371/journal.pone.0109061)
Supplement: Table S1 — DpnI enriches saliva organisms that are known to be involved in oral diseases. DNA isolated from saliva was segregated with DpnI and the DNA used to prepare NGS libraries. The fold of enrichment was calculated based on NGS mapped reads normalized to total reads in the DpnI bound versus input fractions. In the cases where there were no mapped reads in the input fraction (T. denticola) the normalized mapped read counts are listed. (DOC) [file pone.0109061.s006.doc]

**Table S1.** DpnI enriches saliva organisms that are known to be involved in oral diseases. The fold of enrichment was calculated based on NGS mapped reads.

| **Species** | **Fold enrichment** |
| --- | --- |
| *Aggregatibacter actinomycetemcomitans* | 17.14 |
| *Fusobacterium nucleatum* | 6.61 |
| *Prevotella nigrescens* | 8.00 |
| *Prevotella intermedia* | 4.67 |
| *Treponema denticola* | 28.64 |
| *Mycoplasma* spp. | 8.42 |
